# Does resilience predict hospital length of stay after total knee arthroplasty? A prospective observational cohort study

**DOI:** 10.1186/s42836-022-00128-5

**Published:** 2022-07-07

**Authors:** Marie K. March, Alison R. Harmer, Bijoy Thomas, Amy Maitland, Deborah Black, Sarah Dennis

**Affiliations:** 1 Physiotherapy Department, Western Sydney Local Health District, Blacktown Hospital, Marcel Cres, Sydney, NSW 2148 Australia; 2 Sydney School of Health Sciences, Faculty of Medicine and Health, The Susan Wakil Health Building, Western Avenue, University of Sydney, Sydney, NSW 2006 Australia; 3 Department of Orthopaedic Surgery, Blacktown Mt Druitt Hospital, Western Sydney Local Health District, Blacktown Hospital, Marcel Cres, Sydney, NSW 2148 Australia; 4 Hospital in the Home, Western Sydney Local Health District, Building 109 Cumberland Hospital Campus, 5 Fleet Street, North Parramatta, Sydney, NSW 2151 Australia; 5 South Western Sydney Local Health District, Liverpool, NSW 2170 Australia; 6 Ingham Institute for Applied Medical Research, 1 Campbell St., Liverpool, NSW 2170 Australia

**Keywords:** Psychological resilience, Length of stay, Arthroplasty, Mental health, Health services research, Psychology, Total knee replacement

## Abstract

**Background:**

Resilience, or the ability to bounce back from stress, is a key psychological factor that is associated with ongoing functional independence and higher quality of life in older adults in the context of chronic health conditions. Emerging research has explored resilience and patient-reported outcomes after TKA. Our primary aim was to explore the relationship between resilience and acute hospital length of stay after total knee arthroplasty (TKA).

**Methods:**

A prospective observational study recruited 75 participants one month before total knee arthroplasty from two Australian hospitals. Two preoperative psychological measures were used: the Brief Resilience Scale, and for comparison, the Depression, Anxiety and Stress Scale-21 (DASS-21). We collected sociodemographic, medical and surgical details, patient-reported pain, function, fatigue and quality of life one month before TKA. Health service data describing acute hospital length of stay, inpatient rehabilitation use, and physiotherapy occasions of service were collected after TKA. Non-parametric analysis was used to determine any differences in length of stay between those with low or high resilience and DASS-21 scores. Secondary regression analysis explored the preoperative factors affecting acute hospital length of stay.

**Results:**

No significant difference was detected in length of stay between those with a low or a high resilience score before TKA. However, the group reporting psychological symptoms as measured by the DASS-21 before TKA had a significantly longer acute hospital length of stay after TKA compared to those with no psychological symptoms [median length of stay 6 (IQR 2.5) days *vs.* 5 (IQR 2) days, respectively (Mann-Whitney U = 495.5, *P*=0.03)]. Multivariate regression analysis showed that anesthetic risk score and fatigue were significant predictors of length of stay, with the overall model demonstrating significance (χ^2^=12.426, df = 4, *P*=0.014).

**Conclusions:**

No association was detected between the brief resilience score before TKA and acute hospital length of stay after TKA, however, symptoms on the DASS-21 were associated with longer acute hospital length of stay. Preoperative screening for psychological symptoms using the DASS-21 is useful for health services to identify those at higher risk of longer acute hospital length of stay after TKA.

## Background

Resilience is a concept that has been used in various ways in the context of human and social research. Windle [1] identified the three essential ideas within resilience: the presence of significant adversity, resources to offset the effects of adversity, and a positive or neutral outcome, or, in simple terms, “the ability to bounce back from stress” [2]. Resilience was first studied in children experiencing adverse life events, as researchers sought to understand why some young people continue to maintain good health, healthy relationships and societal engagement after trauma, while others struggle in these areas [3]. Evaluation of resilience can be quantitative or qualitative, exploring a range of factors that are protective against undesirable outcomes, including biological factors, individual personality characteristics (*e.g.*, optimism), and environmental resources (*e.g.*, social support), which vary depending on the life-stage of the individual, and the nature of their adversity [4]. Research is now exploring resilience in older people, as they experience and respond to adversity, often experienced as limitations in physical function, and reduced social supports [4–6]. High resilience in older adults is associated with increased physical function, higher self-reported quality of life, increased independence with activities of daily living, even in the context of a variety of chronic health conditions [7–11]. These associations between resilience and health outcomes have particular relevance for people undergoing arthroplasty, as they experience cumulative adversity: age-related changes in physical ability and social supports, pain and limited function from osteoarthritis, and the additional physical and psychological stressors from surgery. Assessing patient-reported resilience before arthroplasty may assist clinicians to identify those who are at risk of sub-optimal outcomes after arthroplasty, who are not identified using current methods. Current methods often focus on a formal diagnosis of depression or anxiety, use of psychoactive medications, use of generic quality of life outcome measures, or diagnosis-specific outcome measures [12–15]. Assessing resilience may be a more sensitive method of identifying those at risk of poor outcomes, as it considers how patients respond to adversity, rather than a narrow identification of specific diagnoses [16].

Few studies have explored resilience in a knee arthroplasty population. Those that have, have focused on the relationship between resilience and patient-reported outcomes in the medium-term after TKA [17–19]. Health services need to consider multiple measures of quality of care, incorporating both patient-reported outcomes, as well as service-based outcomes such as hospital length of stay [20]. Acute hospital length of stay (LOS) is often used as a measure of financial costs, and is used to allocate funding for health services under many funding models [21]. Given the high volumes of TKA procedures performed, the high costs for health services in providing TKA, increasing demands for TKA and commensurate projected increases in costs [22, 23], due consideration needs to be given to acute hospital length of stay (LOS) as a key outcome to measure high-value arthroplasty care.

Therefore, the primary aim of this study was to explore the relationship between preoperative resilience and acute hospital LOS after TKA. We hypothesized that patients with low resilience before TKA would have longer LOS after TKA compared to patients with normal/high resilience.

## Methods

We conducted a prospective, observational study of 75 consecutive patients attending a pre-admission education class between 1st October 2016 and 31st December 2017 at one month before elective primary unilateral TKA for osteoarthritis across one hospital service at two geographical sites. We excluded participants with moderate or severe cognitive impairment or further planned orthopedic surgery within six months. Time and funding constraints limited our sample to participants being discharged within our local health district. The current TKA clinical pathway at our hospital service has a goal of discharge home on the fourth postoperative day with weekend physiotherapy service provision. All eligible participants were invited to participate before their single education class by a study investigator, who provided information, answered questions, and obtained written consent. Baseline measures were collected after consent was obtained on the same day.

### Baseline outcomes

Sociodemographic, anthropometric, and medical history data were extracted from the electronic medical records, including anesthetic risk score.

### Psychological outcomes included

- Resilience measured using the Brief Resilience Scale [2]. This six-item scale measures patient-reported outcomes, with three items reversed scored, and scores tallied and then divided by six to provide a mean score ranging from one to five, five indicating high resilience. A score of less than three is classified as “low resilience” [24].
- Depression, Anxiety and Stress Scale-21 (DASS-21) [25]. This 21-item patient-reported outcome measures the correlated domains of depression, anxiety and stress, with published cut-offs for normal, mild, moderate and severe symptoms in each domain. High scores indicate increased severity of psychological symptoms. We used published cut-off scores to dichotomize the sample into two groups, *i.e*., those who were ‘psychologically well’ or those with psychological symptoms (mild, moderate or severe) in each domain.

### Patient-reported outcomes included

- EuroQoL 5D-5L (EQ-5D-5L) is a health-related quality-of-life measure validated for use in the arthroplasty population [26, 27]. The index score is calculated from responses to five questions and transformed into a score between zero and one. The VAS score is one question asking participants to rank their health today on a scale from zero to 100. High scores on both components are indicative of higher health-related quality of life.
- Western Ontario and McMaster Universities Osteoarthritis Index (WOMAC) subscales for pain (5 items, possible score 0-20) and function (17 items, possible scores 0-68) that are valid and reliable among people with TKA [28]. High scores indicate higher pain or worse function.
- Worst pain in the past 24 hours was measured using the Numerical Pain Rating Scale (NPRS), with possible scores from zero to ten, and higher scores indicative of higher pain [29]. This tool was used in addition to the WOMAC pain measure, given our culturally and linguistically diverse population, to capture the variability in daily pain experience of our participants.
- Fatigue Severity Scale: a nine-item Likert scale where high scores indicate higher levels of fatigue. Responses are summed and then a mean score is calculated, with possible scores from one to seven [30].

### Clinician-reported outcomes included:

- 30-second chair stand test (30CST) as a valid and reliable measure of lower limb strength among arthroplasty populations [31, 32]. The score is measured as the maximum number of repetitions of standing up from sitting that a participant can safely complete in 30 seconds. Higher scores indicate higher strength. We allowed participants to use upper limb support as desired to increase adherence and safety.

### Dependent outcomes

Our primary outcome measure was acute hospital LOS, measured in calendar days. We also collected count data regarding postoperative inpatient complications. These were obtained from the electronic medical records by a study investigator at six weeks after TKA. For data analysis, these were grouped into four categories (Table 1). Furthermore, we collected data on health service use, including inpatient rehabilitation use, and physiotherapy occasions of service.

**Table 1:** Classification of complications

Complication group	Description	Examples
Major complications	Medical or surgical events requiring an increased acuity of care, further procedure, significant change in management priorities	Pulmonary embolism, myocardial infarction
Minor complications	Health issues that did not result in an increase in frequency or acuity of health service delivery, but still required minor changes to existing care provision	Wound infection
Health service complications	Delays in discharge after patient was deemed fit for discharge due to availability of in-home support services	Delayed provision of social services such as domestic assistance
Non-specific complications	Delays in discharge that had no specific attributable cause documented in the medical record	Delays in progress with physiotherapy were identified but with no specific cause documented in the medical record

### Data analysis

Sample size calculations assumed LOS has a Poisson distribution, with a one-day difference between groups based on resilience level (80% power, α= 0.05) and two-tailed statistical testing, resulting in a minimum sample size of 75. We allowed for 25% dropout given our culturally diverse population and aimed to recruit 100 participants. The cohort was dichotomized into groups in two analyses: first based on resilience score, and then on DASS-21 score. LOS and complications data were analyzed using non-parametric testing in SPSS to detect differences between groups. Secondary analysis included generalized linear modelling with Poisson loglinear distribution, and a main effects model was used to explore preoperative factors affecting LOS. Variables included in univariate analysis that had a *P* value <0.2 were retained for multivariate analysis [33]. We have included our model of best fit in multivariate analysis, which was not improved when adjusted for sex and BMI, and variables were excluded such that the significance of the overall model was optimized and collinearity was minimized. Missing data were not imputed.

Ethical approval for the study was obtained from Western Sydney Local Health District Human Research Ethics Committee (approval number: AU RED LNR/16/WMEAD/289).

## Results

We identified 154 people as eligible at pre-admission and 91 participants consented to participate in the study. Sixteen participants were subsequently excluded because of missing baseline data, leaving 75 participants in the final cohort dataset. No significant differences were observed in demographics or baseline outcome data between participants who were included or excluded, with *P*>0.05 for all variables (data not shown). Baseline data are presented for the total group (75 participants) in Table 2, with further details given in Appendix. Our sample achieved a 53% adherence to our hospitals’ clinical pathway of discharge on the fourth day after TKA, and 8% of our sample were discharged to inpatient rehabilitation.

**Table 2:** Baseline characteristics

Characteristic	Total group *n*=75
Demographics	
Age – mean (SD)	68 (8.2)
Sex – *n* (%) female	49 (65%)
Right side TKA – number (%)	42 (56%)
Physical health characteristics	
Body Mass Index – mean (SD)	34.8 (9.0)
Number of medical conditions – mean (SD)	3.9 (2.3)
ASA 1-2 – number (%)	38 (51%)
ASA 3-4 – number (%)	37 (49%)
30-second chair stand test (30CST) – mean (SD)	9.9 (3.7)
Psychological characteristics	
Brief Resilience Score (BRS) – mean (SD)	3.5 (0.8)
DASS-21 Depression – mean (SD)	4.7 (4.3)
DASS-21 Anxiety – mean (SD)	4.3 (4.1)
DASS-21 Stress – mean (SD)	5.9 (4.7)
Patient-reported outcomes	
WOMAC pain subscale – mean (SD)	12.0 (4.2)
WOMAC function subscale – mean (SD)	40.2 (14.8)
Numerical Pain Rating Scale (NPRS) worse pain – mean (SD)	6.7 (2.1)
Fatigue Severity Scale (FSS) – mean (SD)	4.6 (1.7)
EQ-5D-5L index – mean (SD)	0.4 (0.3)
EQ-5D-5L VAS – mean (SD)	67.4 (20.2)

### Primary outcomes

**Fig. 1:**
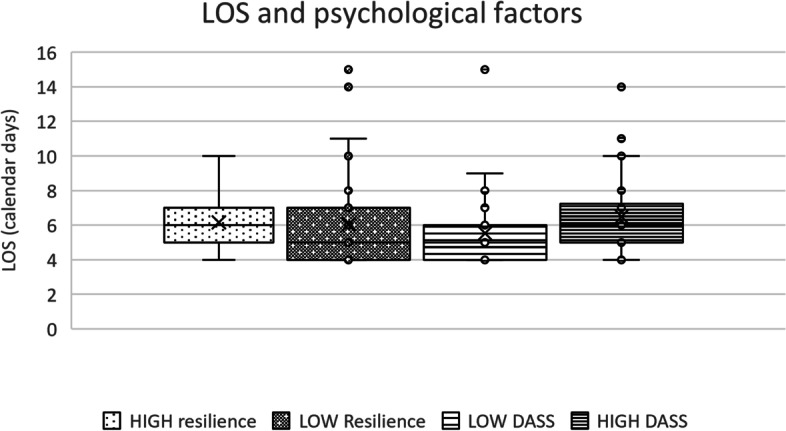
Primary outcomes: LOS and psychological factors

Our first analysis explored the relationship between Brief Resilience Scale score and acute hospital LOS, with subsequent analyses exploring resilience and inpatient rehabilitation use, and physiotherapy occasions of service. Spearman’s correlation between resilience score and LOS detected a weak non-significant relationship (ρ= –0.209, *P*=0.072, 95%CI –0.418 to 0.021). When the cohort was dichotomized using the Brief Resilience score, with the ‘low resilience’ group scoring less than three, and the normal/high resilience group scoring three or above, there was no significant difference detected in LOS between groups based on resilience (Mann-Whitney U=330.0, *P*=0.478). There was no significant difference detected in inpatient rehabilitation use (Mann-Whitney U=305.50, *P*=0.188) or physiotherapy inpatient occasions of service (Mann-Whitney U=344.0, *P*=0.618) (Fig. 1) between the high and low resilience groups.

We repeated this analysis to explore the relationship between DASS-21 scores and LOS. Spearman’s correlation between each dimension of the DASS-21 (depression, anxiety and stress) and LOS showed very low to moderate correlation levels and significant relationships were detected for depression and anxiety (Depression ρ = 0.289, *P *= 0.012, 95%CI 0.063 to 0.487; Anxiety ρ = 0.308, *P *= 0.007, 95%CI 0.083 to 0.503; Stress ρ =0.193, *P*= 0.097, 95%CI –0.037 to 0.404). When the sample was dichotomized using DASS-21 data, using cut-offs for no symptoms in each domain (“psychologically well”) compared with mild, moderate or severe symptoms in any domain (“psychological symptoms”), participants experiencing psychological symptoms in any domain had a significantly longer LOS compared to the psychologically-well group, with greater variation in LOS (median 5 days IQR 2 *vs*. median 6 days IQR 2.5, Mann-Whitney U = 495.5, *P*=0.028). There was a statistically significant difference in inpatient physiotherapy occasions of service between groups (median 4 *vs*. 5 occasions of service, Mann-Whitney U = 487.50, *P *= 0.024); however, no difference in inpatient rehabilitation use was observed (Mann-Whitney U = 671.0, *P *= 0.836) (Fig. 1).

### Secondary outcomes

Table 3 displays the results of the univariate and multivariate analyses. Univariate analysis showed that fatigue, sex, lower limb strength (30CST), anesthetic risk score, worst pain (NPRS) and BMI all had *P* values < 0.2, hence all were included in the initial multivariate model (Table 3). The final most parsimonious multivariate model of factors that predicted LOS (χ^2 ^=14.426, df = 4, *P *= 0.014) was achieved by sequential removal of least significant variables, including age, sex and BMI. In the final model, anesthetic risk score (OR 1.204, 95%CI 1.013–1.432, *P* = 0.035) demonstrated a statistically significant association with LOS, and fatigue (OR 0.939, 95%CI 0.883–1.000, *P* = 0.049) demonstrated a statistically significant association with LOS. No variables included in the multivariate analysis demonstrated collinearity on statistical testing.

**Table 3:** Secondary outcomes: univariate and multivariate analysis of preoperative variables to predict LOS

Baseline variables	Univariate analysis				Final multivariate analysis			
	OR	95% CI OR		***P*** value.	OR	95% CI OR		***P*** value
**Included**								
Fatigue	0.929	0.865	0.998	0.045†	0.939	0.883	1.000	0.049‡
(Intercept)	9.461	1.008	88.768	0.049†	5.022	2.673	9.434	0.000
Sex	1.235	0.985	1.549	0.068†*				
30-sec chair stand test	0.974	0.945	1.004	0.087†	0.974	0.947	1.002	0.064
ASA score	1.203	0.968	1.494	0.095†	1.204	1.013	1.432	0.035‡
Worst pain (NRPS)	1.053	0.988	1.122	0.111†	1.041	0.994	1.090	0.091
BMI	1.009	0.996	1.023	0.168†*				
**Excluded**								
Resilience (BRS)	0.917	0.796	1.058	0.235				
Depression (DASS-21)	1.036	0.976	1.100	0.243				
WOMAC function	0.993	0.980	1.006	0.280				
Stress (DASS-21)	0.973	0.925	1.023	0.280				
Side	0.895	0.724	1.106	0.304				
Anxiety (DASS-21)	0.977	0.931	1.025	0.338				
WOMAC pain	1.014	0.974	1.056	0.493				
Medical conditions	1.013	0.958	1.072	0.652				
EQ-5D-5L	0.999	0.993	1.005	0.699				
Age	0.997	0.981	1.013	0.727				
Socioeconomic status	1.000	0.999	1.001	0.833				

*NPRS* Numerical Pain Rating Scale, *BMI* Body mass index, *BRS* Brief Resilience Scale, *DASS-21* Depression, Anxiety Stress Scale-21

*sex and BMI were excluded on further refinement of the model despite meeting inclusion criteria; † *P* < 0.05 in univariate model; ‡ *P* < 0.05 in multivariate model

## Discussion

Our study has demonstrated that resilience before TKA, using the Brief Resilience Scale, was not associated with acute hospital LOS or inpatient physiotherapy occasions of service after TKA. However, our study demonstrated that mild, moderate or severe symptoms on any domain of the DASS-21 before TKA were associated with a longer LOS and higher inpatient postoperative physiotherapy occasions of service after TKA. Neither psychological measure was associated with inpatient rehabilitation use after TKA. Our secondary regression analysis demonstrated that high levels of fatigue and higher anesthetic risk score before TKA predicted higher LOS after TKA.

Our study demonstrated no association between preoperative resilience and short-term health service outcomes after TKA. In contrast, previous studies have shown that low resilience before TKA was associated with higher pain, reduced satisfaction, and worse patient-reported function after TKA [17–19, 34]. Our findings may indicate the need for a different measure of resilience to detect changes in LOS, such as the Connor-Davidson Resilience Scale [35]. The work of Windle *et al*. [36] has shown that the BRS, although a valid and reliable measure of resilience, does focus on resilience at the level of the individual, without acknowledging the social and environmental factors that influence resilience.

Understanding how individual, social and environmental factors influence resilience may be more useful in predicting TKA outcomes after hospital discharge compared to the inpatient setting. Once patients have left the standardized social and environmental context of an inpatient hospital setting, differences in resilience may have clinically significant effects on TKA outcomes. One mechanism of this effect may be that resilience may influence adherence to postoperative care in the home setting, including exercise [37, 38]. Previous work has shown an association between high resilience in older adults and higher levels of physical function and exercise [39, 40] and that many people do not increase their physical activity levels after TKA, despite improved pain and function [41]. Future research needs to explore whether resilience can predict long-term patient outcomes, such as physical activity, which has important implications for the health of older people beyond their index arthroplasty.

In contrast to our findings on resilience, our study demonstrated that the Depression, Anxiety and Stress Scale-21, used to assess psychological health before TKA, is useful in predicting those at risk of longer acute hospital LOS after TKA. Our study was innovative in using the Depression, Anxiety and Stress Scale-21, which few other studies have used. We have demonstrated that it is feasible for an orthopedic clinician to use in the arthroplasty context. Previous studies have used the Hospital Anxiety and Depression Scale (HADS), a valid and reliable measure that uses a two-factor model to assess depression and anxiety symptoms [42]. The DASS-21 provides a three-factor model that captures stress as a separate factor, characterized by tension, irritability, and the inability to relax, which has been outlined in the psychological literature [43]. Given the consistent positive correlations between psychological factors and physiological stress in the perioperative period [44], incorporating stress into preoperative psychological screening is a justified choice. The DASS-21 may be a more sensitive screening tool than the HADS to use before TKA to identify those at risk of sub-optimal postoperative outcomes.

There is a common perception that, although some patients report psychological distress before arthroplasty, this distress resolves postoperatively as pain, function and quality of life improve, and therefore does not warrant particular intervention in the perioperative period [45]. However, our study is consistent with many published studies that show that preoperative psychological factors are associated with worse outcomes for patients and health services after TKA, which are not necessarily equated with a formal psychological diagnosis [46–48]. For patients, these outcomes include pain, function and satisfaction [49–51]. For health services, these outcomes are often used as measures to determine quality of care, including hospital LOS and complications [13, 48, 52]. Applying best practice models of high quality, patient-centred health care in the arthroplasty context requires clinicians to identify and address psychosocial concerns of patients, rather than assuming postoperative resolution [20, 53]. The presence of psychological symptoms should not be used as a justification to withhold access to TKA, given that significant improvements can be made in pain and function [54, 55] but needs to be viewed as an opportunity for preoperative care that addresses physical and psychological health, in line with emerging evidence [56–58]. We recommend that arthroplasty surgeons, and their care teams, incorporate formal assessment of psychosocial factors as a part of standard preoperative assessment. More broadly, our evidence supports the assertion that best practice arthroplasty care should follow the biopsychosocial approach to patient care shown to be effective in osteoarthritis management, as a way of ensuring TKA remains a high-value procedure for all patients and health services, including patients with complex psychosocial needs [59, 60] This would allow for individualized care, including advice and education, exercise and physical activity, optimized analgesia, and weight management, underpinned by patient-centred care principles [61]. Current models of osteoarthritis care led by physiotherapists are effective and scalable [61] and future research needs to establish how this biopsychosocial approach can be implemented in the perioperative care setting.

Our secondary analysis, although limited by sample size, indicates that increased anesthetic risk scores and higher fatigue levels before TKA are associated with longer LOS after TKA. Our findings that high anesthetic risk score influences LOS is consistent with previous work [62]. Fatigue is a complex phenomenon, experienced by people with a wide range of chronic diseases and it incorporates disease-specific and psychosocial factors [63]. It is more highly correlated with the number of chronic diseases experienced rather than the severity of chronic disease [63, 64]. The majority of our TKA cohort evidenced multimorbidity, yet few studies have explored fatigue in the TKA population, despite relatively high prevalence in people with osteoarthritis [64, 65]. Hodges and colleagues found higher fatigue levels were associated with reduced physical activity and poorer patient-reported outcomes in the medium term after TKA [41, 66]. Taking a biopsychosocial approach recommended above ensures that fatigue is identified and mitigated before it adversely impacts TKA outcomes for patients. Future research is needed to explore how to optimize both physical and psychosocial health status in order to reduce the risk of longer LOS after TKA.

There is an increasing number of studies exploring interventions that optimize outcomes after TKA. Preoperative education before TKA, although widely used clinically, has been shown to be ineffective in changing postoperative outcomes after TKA for most people [67]. There are mixed results on the effect of preoperative exercise therapy on postoperative outcomes [68–70]. However, one study demonstrated that individualized allied health interventions targeting patients with complex needs were an effective approach for optimizing postoperative outcomes [71]. A review by Sorel *et al*. found that various perioperative psychological interventions were effective in improving outcomes after TKA, however, the quality of evidence was low to moderate [57]. A majority of psychological interventions in that study were based on cognitive-behavioral methods, which achieved mixed results [72, 73], however, positive effects on TKA outcomes have been shown with mindfulness-based therapy [56, 74]. Mindfulness is a key aspect of Acceptance and Commitment Therapy [75] and this psychological approach has been shown to be more effective in older adults with chronic pain than cognitive-behavioral therapy [76]. Given the older age of the TKA population and early positive results with use of mindfulness, future research needs to explore TKA care options informed by Acceptance and Commitment Therapy [77, 78].

Strengths of our study include the prospective collection of data, in a real-world clinical setting, by clinicians already embedded in the health service. Our study is one of the few exploring the concept of resilience in the arthroplasty population. We have also used the DASS-21, a more comprehensive measure of psychological symptoms. Limitations of our study are centred around sample size, including the number of participants who were willing to be recruited. Our study was not powered to adequately assess our secondary outcomes, and hence limited the scope of our multivariate regression analyses. Our population is culturally and linguistically diverse, with low health literacy, which may explain why our prospective study has a relatively low recruitment rate and higher drop-out rate compared to other retrospective registry studies, which limited any secondary analysis of follow-up data. Only 8% of our sample was transferred to inpatient rehabilitation, which may limit our ability to draw conclusions regarding preoperative predictors for this outcome.

## Conclusion

Routine assessment of psychological symptoms before TKA using the DASS-21 scale can identify those at risk of longer LOS who will likely require more inpatient physiotherapy following TKA. A patient-centred approach to TKA clinical pathways integrating assessment and management of both physical and psychosocial health is likely to be the best approach to optimize outcomes for all TKA patients.

## Data Availability

The datasets generated and/or analyzed during the current study are not publicly available due to legislation regarding privacy of health information in our jurisdiction but de-identified data are available from the corresponding author on reasonable request.

## Appendix

**Table:**

Characteristics	Total group *n*=75	Low resilience *n*=12	Normal/high resilience *n*=63	No psych symptoms *n*=34	Presence of psych symptoms *n*=41
Demographics					
Age – mean (SD)	68 (8.2)	68 (10.2)	68 (7.8)	66.2 (6.7)	68.7 (9.0)
Sex – *n* (%) female	49 (65%)	10 (83%)	39 (62%)	21 (43%)	28 (57%)
Right side TKA – number (%)	42 (56%)	9 (21%)	33 (79%)	16 (38%)	26 (62%)
Physical health characteristics					
Body Mass Index – mean (SD)	34.8 (9.0)	35.8 (9.6)	34.7 (8.9)	32.6 (8.5)	36.7 (9.0)
Number of medical conditions – mean (SD)	3.9 (2.3)	4.9 (2.9)	3.7 (2.1)	2.9 (1.9)	4.7 (2.3)
ASA score – mean (SD)	2.48 (0.58)	2.50 (0.67)	2.48 (0.56)	2.26 (0.57)	2.66 (0.53)
30-second chair stand test (30CST) – mean (SD)	9.9 (3.7)	8.3 (3.3)	10.2 (3.7)	10.5 (3.5)	9.3 (3.8)
Psychological health characteristics					
Brief Resilience Score (BRS) – mean (SD)	3.5 (0.8)	2.4 (0.4)	3.7 (0.6)	3.7 (0.8)	3.4 (0.7)
DASS-21 Depression – mean (SD)	4.7 (4.3)	7.8 (6.1)	4.0 (3.6)	1.29 (1.2)	7.5 (3.8)
DASS-21 Anxiety – mean (SD)	4.3 (4.1)	7.2 (6.4)	3.7 (3.3)	1.5 (1.2)	6.6 (4.2)
DASS-21 Stress – mean (SD)	5.9 (4.7)	9.6 (6.7)	5.2 (3.9)	2.3 (2.1)	8.9 (4.1)
Patient-reported measures					
WOMAC pain subscale – mean (SD)	12.0 (4.2)	13.6 (4.2)	11.7 (4.2)	10.5 (3.8)	13.1 (4.2)
WOMAC function subscale– mean (SD)	40.2 (14.8)	46.4 (12.5)	39.0 (15.0)	36.2 (13.6)	43.5 (15.1)
Numerical Pain Rating Scale (NRPS) worse pain – mean (SD)	6.7 (2.1)	7.1 (1.4)	6.6 (2.3)	6.2 (2.3)	7.1 (1.9)
Fatigue Severity Scale (FSS) – mean (SD)	4.6 (1.7)	4.9 (1.7)	4.5 (1.7)	3.9 (1.8)	5.2 (1.2)
EQ-5D-5L index – mean (SD)	0.4 (0.3)	0.39 (0.30)	0.45 (0.31)	0.56 (0.20)	0.33 (0.34)
EQ-5D-5L VAS – mean (SD)	67.4 (20.2)	58.6 (22.4)	69.0 (19.5)	75.4 (17.4)	60.7 (20.1)

## Abbreviations

LOS: Length of stay
BRS: Brief Resilience Scale
DASS-21: Depression, Anxiety and Stress Scale-21
WOMAC: Western Ontario McMaster Osteoarthritis Index
HADS: Hospital Anxiety and Depression Scale
30CST: 30 second chair stand test
NPRS: Numerical Pain Rating Scale
BMI: Body Mass Index
ASA: American Society of Anesthesiologists physical status classification, *i*.*e*. Anesthetic risk score
